# Supplementary material for: ‘What I cannot create, I do not understand’: functionally validated synergism of metabolic and target site insecticide resistance
Source: Proc Biol Sci. 2020 May 27;287(1927):20200838. doi: 10.1098/rspb.2020.0838 (PMC7287358; doi:10.1098/rspb.2020.0838)
Supplement: Tables and figures [file rspb20200838supp1.pdf]

Supplementary Information

Table S1: *Drosophila* strains used in this study

Strain	Description	Source
y^1 M{nos-Cas9.P}ZH-2A w* (referred as nos.Cas9)	Expresses Cas9 in germ line under *nanos* promoter	Bloomington Drosophila Stock Center #54591 [19]
yw; TM3 *Sb e*/TM6B *Tb Hu e*	Balancers for 3rd chromosome (TM3 with *Stubble* marker. TM6B with *Tubby* and *Humoral* markers)	IMBB flyroom stock
yw; *Cy*O/*Sco*	Balancer (CyO) for 2nd chromosome, marked with *Curly*.	
(FRT)w^+ / FM7c *Hw w B*	Balancer (FM7c) for X chromosome marked with *Bar*	
FM7 ; *Cy*O / *Sp*	Balancer (FM7c) for X chromosome marked with *Bar*; balancer for 2nd chromosome (CyO) marked with *Curly*, non-balancer 2nd chromosome marked with *Sternopleural*	
yw nos.int; attP40	Expresses Phic31 integrase under *nanos* promoter; contains attP40 landing site in 2nd chromosome, *yw* genetic background	[18]
UAS-CYP9J28	Bears a *Cyp9j28* transgene under UAS at random P-element insertion point at 2nd chromosome, *yw* genetic background.	[17]
HR-GAL4	Bears HR-GAL4 transgene (marked with miniwhite) at P-element insertion site in 2nd chromosome	[10]
para^{L1014F}	Bears a L1014F mutation in *para;* X-chromosome derived from nos.Cas9, other chromosomes from *yw* background.	This study
para^{V1016G}	Bears a V1016G mutation in *para;* X-chromosome derived from nos.Cas9, other chromosomes from *yw* background.	
*yw;*HR-GAL4> UAS-CYP9J28(2N)	Bears both HR-GAL4 and UAS-CYP9J28 at the 2nd chromosome, derived from genetic crossover, other chromosomes from *yw* background.	
UAS-CYP6BQ23	Bears a *Cyp6bq23* transgene under UAS at the attP40 insertion point at 2nd chromosome, *yw* genetic background.	
*yw;*attP40	Contains empty attP40 landing site in 2nd chromosome, *yw* genetic background (same as UAS-CYP6BQ23 but without the transgene)	
para^{L1014F};HR-GAL4	X-chromosome derived from *para*^{L1014F}, 2nd chromosome from HR-GAL4, other *yw*	
para^{L1014F};UAS-CYP6BQ23	X-chromosome derived from *para*^{L1014F}, 2nd chromosome from UAS-CYP6BQ23, other *yw*	
para^{V1016G};HR-GAL4> UAS-CYP9J28(2N)	X-chromosome derived from *para*^{V1016G}, 2nd chromosome from *yw*;HR-GAL4>UAS-CYP9J28(2N), other *yw*	

Table S2: Primers used in this study

Primer Name	Sequence (5' – 3')	Use	Reference
935F	CTTCGCAGGTCGCCATCCGGAAAG	sgRNA935	This study
935R	AAACCTTTCCGGATGGCGACCTGC		
406F	CTTCGACTTGTAACCGATGTTTAC	sgRNA406	
406R	AAACGTAAACATCGTTTACAAGTC		
205F	CTTCGCCAATTCGATTGAAGGCCT	sgRNA205	
205R	AAACAGGCCTTCAATCGAATTGGC		
kdrF	TCGCTCCAAATCCAACTGAT	Sequencing, allele screening	
kdrR	ACCGACTTTATGCACAGCTT		
ParaInF	GTTTGATTTGCTGTCAAGCC	Sequencing *para* genomic region	
ParaInR	CTGGCATTTTAACTTGTAACACAC		
exoF	AATTTGCCATGTGGGAGTTGC		
exoR	GATCGATCGCATCCTCAACT		
seq_F	CGAAGGTGCGGCAAGTAAC		
seq_R	GTGGCTTGAATGGCTGAGTC		
1014UP	ACCGCTTTCCCGACGGAGAT	Allele screening	
1014DOWN	GCATTATAGCCAGTAAGCATCGTA		
Cyp6bq23_RT_F	AAGTGGAAGAGCTTGAGGGC	RT-PCR	
Cyp6bq23_RT_R	CTCGGGGTTCTTGATGCTGT		
Cyp6bq23_q_F	TCTACGAGAACCCCGAGAAGTTC	qPCR	
Cyp6bq23_q_R	CTTGCTCAGGGTGATGTTGTAGTTC		
Cyp9j28_RT_F	CTCCACGTTCATTCAGACGCT	RT-PCR	[17]
Cyp9j28_RT_R	CTCGAGTTCCCAAATACCTGC		
RPL11_Dm_F	CGATCCCTCCATCGGTATCT	RT-PCR qPCR	[10]
RPL11_Dm_R	AACCACTTCATGGCATCCTC		
RPL32_Dm_F	AGCATACAGGCCCAAGATCG	qPCR	[38]
RPL32_Dm_R	GCACCAGGAACTTCTTGAATCC		
pPel_uas F	GAAGAGAACTCTGAATAGGGAATTG	Sequencing	[26]
pPel_sv40 R	CAAATGTGGTATGGCTGATTATG		

Table S3: Contact bioassay deltamethrin responses of transgenic flies expressing pyrethroid metabolizing P450s alone or along engineered target site resistance mutations in their voltage gated sodium channel (*para*)

Strain/Cross	LC_{50} (µg/vial)	(95% FL)	Slope (±SE)	RR (vs HR-GAL x yw.attP40)	RR (vs nos. Cas9)
HR-GAL4 x *yw*; attP40	1.689	(0.65-2.61)	1.406(±0.270)	1	
HR-GAL4 x UAS-CYP6BQ23	16.35	(9.15 – 25.9)	2.455(±0.388)	9.68	
[1]HR-GAL4>UAS-CYP9J28(2N)	4.249	(2.83- 6.814)	2.9(±0.33)	2.52	
nos.Cas9	5.45	(2.4- 8.57)	2.748(±0.514)	3.23	1
para^{L1014F}	866.02	(746.4 – 1,029.2)	6.895(±1.063)	512.74	158.9
para^{V1016G}	295.567	(222.252-440.5)	2.27(±0.42)	174.99	54.23
[2]*para*^{L1014F} ; HR-GAL4> UAS-CYP6BQ23	>5,000	n/a		**>2960**	**>917**
[3]*para*^{V1016G} ; HR-GAL4>UAS-CYP9J28(2N)	2441.675	(1611.612-3816.105)	2.67(±0.44)	1445.63	448.13

[1]: homozygous recombinant *yw*; HR-GAL4>UAS-CYPJ28(2N) contains two copies of driver and responder

[2]: *para*^{L1014F} ; HR-GAL4 x *para*^{L1014F} ; UAS-CYP6BQ23

[3]: *para*^{V1016G} ; HR-GAL4>UAS-CYPJ28(2N) contains two copies of driver and responder in *para*^{V1016G} X-chromosome background

Table S4: P-values of One-Way ANOVA test (Figure 3) for pupation time (Day 7 above diagonal, Day 8 below diagonal) among different genotypes of "Beetle" and "Mosquito" allelic combinations. Significant values are shown in bold.

**Beetle**	*para*^{L1014F}	nos.Cas9	*yw*;HR-GAL4>UAS-CYP6BQ23	*para*^{L1014F};HR-GAL4>UAS-CYP6BQ23	HR-GAL4>*yw*;attP40
para^{L1014F}		0.9932	0.7676	**0.0044**	0.8876
nos.Cas9	0.9987		0.9590	**0.0249**	0.9915
yw;HR-GAL4>UAS-CYP6BQ23	0.9148	0.9845		0.0896	0.9991
para^{L1014F};HR-GAL4>UAS-CYP6BQ23	**0.0274**	0.0807	0.1829		0.0504
HR-GAL4>*yw*;attP40	0.9583	0.9957	0.9999	0.1338	
**Mosquito**	*para*^{V1016G}	nos.Cas9	*yw*;HR-GAL4>UAS-CYP9J28(2N)	*para*^{V1016G};HR-GAL4>UAS-CYP9J28(2N)	HR-GAL4>*yw*;attP40
para^{V1016G}		0.9802	0.8092	**0.0110**	>0.9999
nos.Cas9	0.9993		0.9896	**0.0039**	0.9869
yw;HR-GAL4>UAS-CYP9J28(2N)	0.4952	0.6988		**0.0004**	0.8399
para^{V1016G};HR-GAL4>UAS-CYP9J28(2N)	**0.0048**	**0.0045**	**<0.0001**		**0.0093**
HR-GAL4>*yw*;attP40	0.9995	0.9920	0.3678	**0.0089**	

Table S5: P-values of One-Way ANOVA test (Figure 3) for total oviposition among different genotypes of "Beetle" and "Mosquito" allelic combinations. Significant values are shown in bold.

**Beetle**	*para*^{L1014F}	nos.Cas9	*yw*;HR-GAL4>UAS-CYP6BQ23	*para*^{L1014F};HR-GAL4>UAS-CYP6BQ23	HR-GAL4>*yw*;attP40
para^{L1014F}					
nos.Cas9	**0.0001**				
yw;HR-GAL4>UAS-CYP6BQ23	**<0.0001**	0.9482			
para^{L1014F};HR-GAL4>UAS-CYP6BQ23	**0.0008**	>0.9999	0.7084		
HR-GAL4>*yw*;attP40	**<0.0001**	0.8735	>0.9999	0.5623	
**Mosquito**	*para*^{V1016G}	nos.Cas9	*yw*;HR-GAL4>UAS-CYP9J28(2N)	*para*^{V1016G};HR-GAL4>UAS-CYP9J28(2N)	HR-GAL4>*yw*;attP40
para^{V1016G}					
nos.Cas9	0.4008				
yw;HR-GAL4>UAS-CYP9J28(2N)	0.6614	**0.0067**			
para^{V1016G};HR-GAL4>UAS-CYP9J28(2N)	**0.0003**	**<0.0001**	**0.0436**		
HR-GAL4>*yw*;attP40	**0.0087**	0.8596	**<0.0001**	**<0.0001**	

>vgscL1014F (2080 bp)
gtgtattatgtgtgtgttctgttcactaaatgtacgtattagtaaacgtaacgtattcgtgtctatgcgtcctgggaaccattgtaatgccaatttatttagacccacctgtctgtatgtactgctattgcgtttgtgtttgtg
ttcaccaaactcggaatcggaatccaaatggattgcggacaactgaaagctatgccatccatttggattccttcgagacagatataaccatgctaccttataatttctttcacacccacattgagaattcgttacttttgtggc
ctgcttagctcaccagtccgtgtatccgaataaaccaagccgccgcccctcctaagcaatccagcatagtcgatatctagcgactataagaatggtttatgatttacttggagctcaagatctttggatcattgagtgcccgt
gtcctccgtaagtacacctgccagcgaaggtgcggcaagtaacttttctcctagctaatttactccaagctcgtctatttgattttttttccttcctaagcattttgtatatgcgatcgcaagcgtaaactttgtttagctac
acactttcgtagctctatagcctgaagaactcgataaaatcaaatgatgttgatgcagatgaggcgtgcgatactttttggttcactcttttttttttttgtgtacgatttttctggcattttaacttgtaacacaccgttttt
ttgtgagtgctcgctgcgctgaacaccgtttgcacacgcttttgtatgtggcttgacagcaaatcaaaccaaaccaaaaaaaaaaaaatcaattaaaacaattgaaactgtctccaaatatccccaaaaaactataaactatact
cgtagaaaacgaagcgagctagaaactattgctaattaatgccaaaccatcgacagATCACAAGGACCGCTTTCCCGACGGAGATCTGCCGCGCTGGAACTTCACCGACTTTATGCATAGCTTCATGATCGTGTTCCGGGTGCT
CTGCGGAGAATGGATCGAGTCCATGTGGGACTGCATGTACGTGGGCGATGTCTCGTGCATTCCCTTCTTCTTGGCTACCGTTGTCATCGGCAATTTTGTGgtgagtactcttatcgaactgctgacttgtatacgatgcttact
ggctataatgctgacttatcgcctctaaccatggctttaagcatcgcattgtagatgtagataactaaattgtgatcgtatatttgcagGTACTTAACCTTTTCTTAGCCTTGCTTTTGTCCAATTTTGGCTCATCTAGCTTAT
CAGCGCCGACTGCCGATAACGATACGAATAAAATAGCCGAGGCCTTCAATCGAATTGGCCGATTTAAAAGTTGGGTTAAGCGTAATATTGCTGATTGTTTCAAGTTAATACGTAACAAATTGACAAATCAAATAAGTGATCAAC
CATCAGGTGAGAGGACCAACCAGATCAGTTGGATTTGGAGCGAAGgtttttatccacttgttttccaattactaaactacttttaattatatgatactcgtaatactcgtatattgccaacgatataattgcgtataccgttaa
cgttaacttcaactcactattggccagtaattgaattgcgtaatcgatctaaaaccaaatatcagtatctaaatcaattgcaaataccaaaaatcaaatcaaacccaattcgcattctgtatactaattgtaaaaggaaatatc
ccccaaaaactaaaaatacgttcttagctgacaatttgcaaattctctttagttagtttcaagtatacctcttcccccgccctctagacctagacctagaccttttaacctcgacctcagcctcagactcagccattcaagcca
ctcaccagaacatgtatctgtaaagccaaaaagtattcatcctggtattcatattcgatcgagagattgcttccatttctttggtttgtttgtatcggttcccgttcgatttgaacctaagtgttgttgttcttcagccggtca
gtgatcctctgaactctgacctcagacctcagacctcagacctcagacctcagacctgagacct

>vgscV1016G (2160 bp)
tcggaatccaaatggattgcggacaactgaaagctatgccatccatttggattccttcgagacagatataaccatgctaccttataatttctttcacacccacattgagaattcgttacttttgtggcctgcttagctcaccag
tccgtgtatccgaataaaccaagccgccgcccctcctaagcaatccagcatagtcgatatctagcgactataagaatggtttatgatttacttggagctcaagatctttggatcattgagtgcccgtgtcctccgtaagtaca
cctgccagcgaaggtgcggcaagtaacttttctcctagctaatttactccaagctcgtctatttgattttttttccttcctaagcattttgtatatgcgatcgcaagcgtaaactttgtttagctacacactttcgtagctct
atagcctgaagaactcgataaaatcaaatgatgttgatgcagatgaggcgtgcgatactttttggttcactcttttttttttttgtgtacgatttttctggcattttaacttgtaacacaccgtttttttgtgagtgctcgctg
cgctgaacaccgtttgcacacgcttttgtatgtggcttgacagcaaatcaaaccaaaccaaaaaaaaaaaaatcaattaaaacaattgaaactgtctccaaatatccccaaaaaactataaactatactcgtagaaaacgaagcg
agctagaaactattgctaattaatgccaaaccatcgacagATCACAAGGACCGCTTTCCGGATGGCGACCTGCCGCGCTGGAACTTCACCGACTTTATGCACAGCTTCATGATCGTGTTCCGGGTGCTCTGCGGAGAATGGATC
GAGTCCATGTGGGACTGCATGTACGTGGGCGATGTCTCGTGCATTCCCTTCTTCTTGGCCACCGTTGTCATCGGCAATCTTGTGgtgagtactcttatcgaactgctgacttgtaaacgatgtttacttgctataatgctgact
tatcgcctctaaccatggctttaagcatcgcattgtagatgtagataactaaattgtgatcgtatatttgcagGGACTTAACCTTTTCTTAGCCTTGCTTTTGTCCAATTTTGGCTCATCAAGCTTATCAGCCCCGACTGCCGA
TAACGATACGAATAAAATAGCTGAGGCCTTCAATCGAATTGGCCGATTTAAAAGTTGGGTTAAGCGTAATATTGCTGATTGTTTCAAGTTAATACGTAACAAATTGACAAATCAAATAAGTGATCAACCATCAGGTGAGAGGAC
CAACCAGATCAGTTGGATTTGGAGCGAAGgtttttatccacttgttttccaattactaaactacttttaattatatgatactcgtaatactcgtatattgccaacgatataattgcgtataccgttaacgttaacttcaactca
ctattggccagtaattgaattgcgtaatcgatctaaaaccaaatatcagtatctaaatcaattgcaaataccaaaaatcaaatcaaacccaattcgcattctgtatactaattgtaaaaggaaatatcccccaaaaactaaaaa
tacgttcttagctgacaatttgcaaattctctttagttagtttcaagtatacctcttcccccgccctctagacctagacctagaccttttaacctcgacctcagcctcagactcagccattcaagccactcaccagaacatgta
tctgtaaagccaaaaagtattcatcctggtattcatattcgatcgagagattgcttccatttctttggtttgtttgtatcggttcccgttcgatttgaacctaagtgttgttgttcttcagccggtcagtgatcctctgaactc
tgacctcagacctcagacctcagacctcagacctcagacctgagacctcctctgaccaatgtgagtcgcactgagggaactgtgccgtgccgtgccatttccgatcacatcatacccaacaaacaactaaaacaaatcgtaacc
gtaaccgtatccgtcataatctttagcattacttagatagtctgtatttagctgaaacgaaagtaacagtatcagtaccagtaccagtaatccaaaaaccaagtatattgtactcttatacgtattttccttagatacatatta

>CYP6BQ23 (1585 bp)
ACGCGT*CACC***ATG**GTGCTGATCACCAACACCCTGACCTACGACCTGGGCATCTTCCTGGCCACCCTGATCACCGCCTTCGTGATCTACATCAAGTGGGTGTACACCTACTGGCAGAAGAAGGGCCTGGCCACCGAGCCCACCGT
GATCCCCTTCGGCAACGGCGCCAAGGTGGCCACCTGCCAGCAGAACATCGGCGAGCTGTTCAGCGACATGTACTTCAAGTTCAAGAGCAAGGGCCTGAAGCACGGCGGCGCCTACTTCCTGATGAAGCCCTTCTACGTGCCCGT
GGACCTGGAGATCGTGAAGGGCATCATGCAGAACGACTTCGGCAACTTCGTGAACAGGGGCATGTACGTGAACGAGAAGGCCGACACCATGAGCACCAACCTGTTCAACCTGGAGGGCGGCAAGTGGAAGAGCTTGAGGGCCAA
GCTGAGCCCCACCTTCACCAGCGGCAAGCTGAAGATGATGTTCAACACCCTGAGCGAGTGCGGCCTGGGCCTGAACGACATGATCGAGGAGTACGCCAGGACCGGCGAGGAGCTGAACATCAAGGACATCCTGCAGAGGTTCAG
CATCGACATCATCGGCAGCGTGAGCTTCGGCATCGAGTGCAACAGCATCAAGAACCCCGAGAGCGAGTTCTTCCTGAACGGCAAGAGGCTGTTCAGCCTGGAGTTCACCAACATCCTGAGGTTCCTGAGCCCCATCGCCCTGCC
CCACAGCGTGCTGAAGTTCTTCAACTTCAACAGCTTCAACGTGAGCGTGACCAACTTCTTCGACAACATCATCAAGCAGAACGTGGAGTACAGGGAGAAGAACAACATCACCAGGCCCGACTTCTTCCACCTGCTGCTGCAGCT
GAAGAACAGGGGCAAGGTGTGCGACGACGACAAGCTGCAGGACACCAACAAGAGCACCAAGGAGAGCGCCCTGACCCTGGACGAGCTGACCGCCCAGAGCTTCGTGTTCTTCATCGCCGGCTACGAGACCACCAGCACCACCAT
GCCTTCGCCCTGAGCGAGCTGGCCCTGAACCAGGAGATCCAGGAGAAGGCCAGGAAGGAGATCGAGATGGTGGCCGCCAAGCACAACGGCGAGCTGAGCTACGAGGCCATCAAGGACATGACCTACATGGAGCAGATCATCAAC
GAGACCCTGAGGAAGTACCCCCCCGTGCCCATCCTGCTGAGGAAGTGCAACAAGACCTACCCCGTGCCCGGCACCGACGTGATCATCGAGAAGGACGACATGGTGGCCATCAGCTGCCTGGCCATCCAGAACGACCCCGAGATC
TACGAGAACCCCGAGAAGTTCGACCCCGACAGGTTCAGCGCCAAGAACACCGCCAGCAGGCACCAGTTCGCCCACATCCCCTTCGGCGAGGGCCCCAGGATCTGCATCGGCATGAGGTTCGCCCTGATGGAGGCCAAGGTGGGC
ATGGCCGCCATCCTGAAGAACTACAACATCACCCTGAGCAAGAGGACCAAGGTGCCCATCCAGCTGGACCCCAAGAGCTTCATCAGCGCCCCCAAGGACGGCATCTGGATCCACGCCAAGAGGATCAGCACCGAC**TAA**CTCGAG

Figure S1: Sequences of donor/expression plasmids synthesized for use in this study.

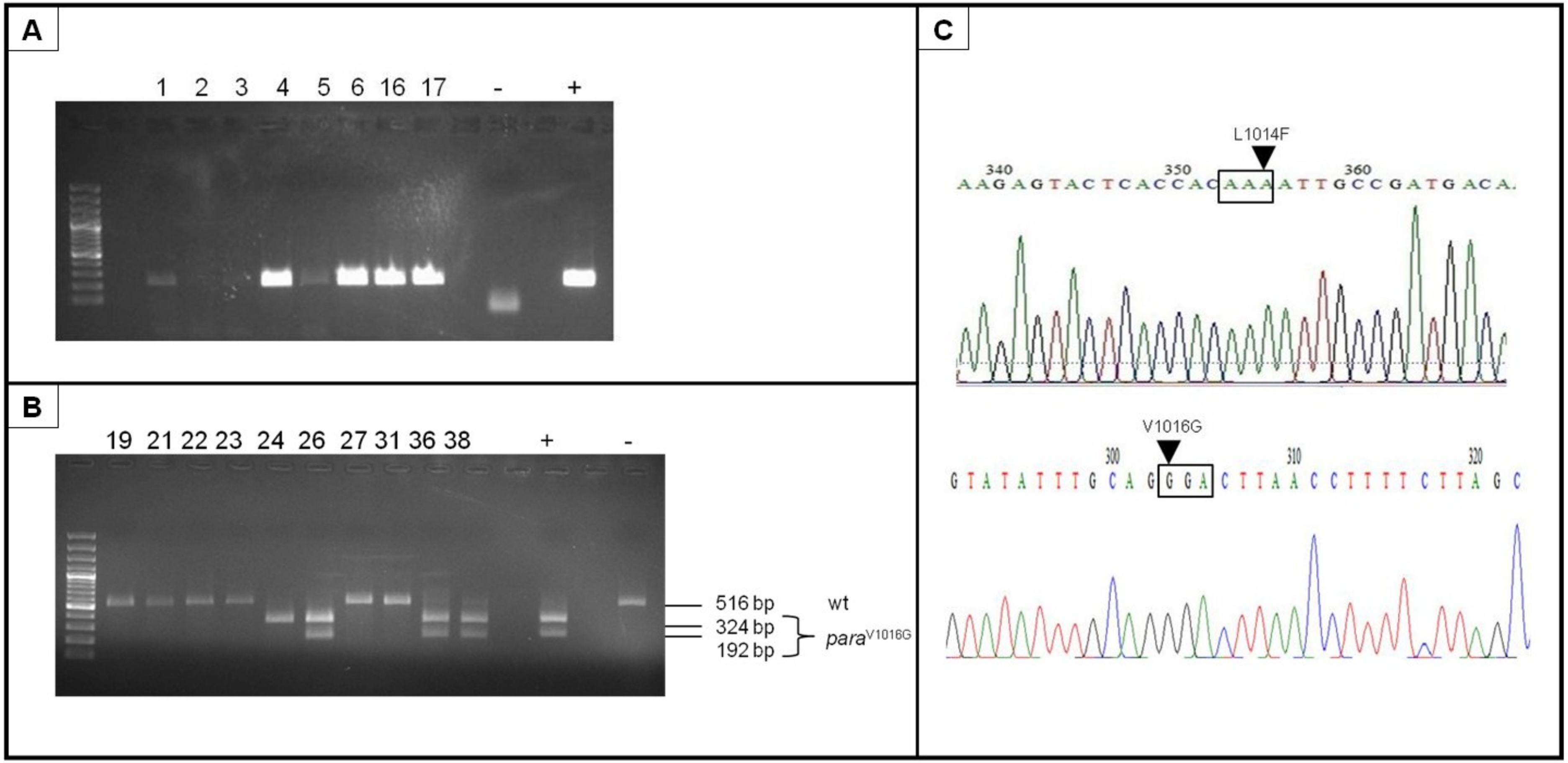
A
1 2 3 4 5 6 16 17 - +
B
19 21 22 23 24 26 27 31 36 38 + -
516 bp
324 bp
192 bp
wt
paraV1016G
C
L1014F
340 350 360
AAGAGTACTCACCACAAAATTGCCGATGACA.
V1016G
300 310 320
GTATATTTGCAGGGACTTAACCTTTTCTTAGC

Figure S2: Screening for genome-modified flies. **A**: PCR screening following digestion with MscI of template DNA from pools of G_1 flies derived from different G_0 (injected) individuals using a specific primer pair (1014UP/1014DOWN) for L1014F mutation [-: nos.Cas9 DNA (negative control), +: donor plasmid template (positive control)]. **B**: Screening of G_1 flies for a *para*^{V1016G} allele; following digestion with HaeII, PCR amplification with a "generic" primer pair (kdrF/kdrR), and digestion of 516 bp product with HindIII. The wild-type allele remains uncut, while the genome modified *para*^{V1016G} allele is cut in two smaller bands; three positive crosses are visible [-: nos.Cas9 DNA (negative control), +: donor plasmid template (positive control)]. **C**: Sequencing of the relevant *para* region in homozygous genome modified flies. Top: L1014F (reverse complement), Bottom: V1016G.

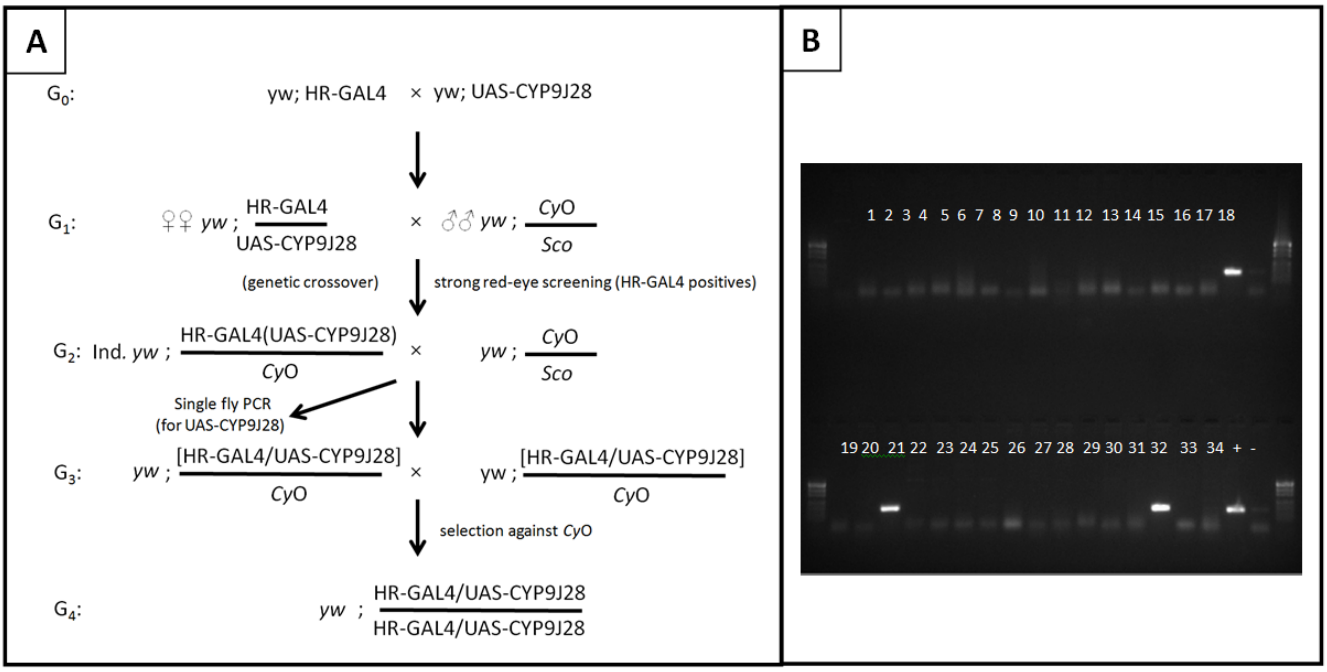

Figure S3: Generation of homozygous recombinant strain *yw;*HR-GAL4>UAS-CYP9J28(2N). **A**: Crossing scheme for the generation of a strain bearing both HR-GAL4 and UAS-CYP9J28 in the 2nd chromosome, following a cross between lines HR-GAL4 [10] and UAS-CYP9J28 [17] that produces a heterozygous genotype (*y*)*w*;HR-GAL4>UAS-CYP9J28. Since both strains were originally generated by P-element mediated transgenesis at random (unknown) positions at the 2nd chromosome, the frequency of genetic crossover is proportional to the distance between the relevant insertion positions. Virgin (*y*)*w*;HR-GAL4/UAS-CYP9J28 females were crossed with *yw*;*Cy*O/*Sco* balancer males and the *Cy* progeny was monitored for the characteristic red-eye phenotype (derived from the w^+ marker expression marking the HR-GAL4 transgene) in contrast to orange-eye phenotype (derived from the miniwhite marker expression marking the UAS-CYP9J28 transgene). Selected individuals, expected to have the HR-GAL4 transgene opposite to a *Cy*O balancer, were crossed to *yw*;*Cy*O/*Sco* balancer flies and after giving progeny they were individually screened for the presence of the UAS-CYP9J28 transgene by PCR amplification (B). Heterozygous *yw*; [HR-GAL4_UAS-CYP9J28] / CyO flies

were then intercrossed to give the homozygous *yw*; HR-GAL4>UAS-CYP9J28(2N) strain following selection against *Cy* marker. **B**: Single fly PCR genotyping for the presense of the UAS-CYP9J28 transgene using primer pair Cyp9j28_RT_F/R. The presence of the diagnostic fragment indicates a genetic recombination event in three out of the 34 individuals examined.

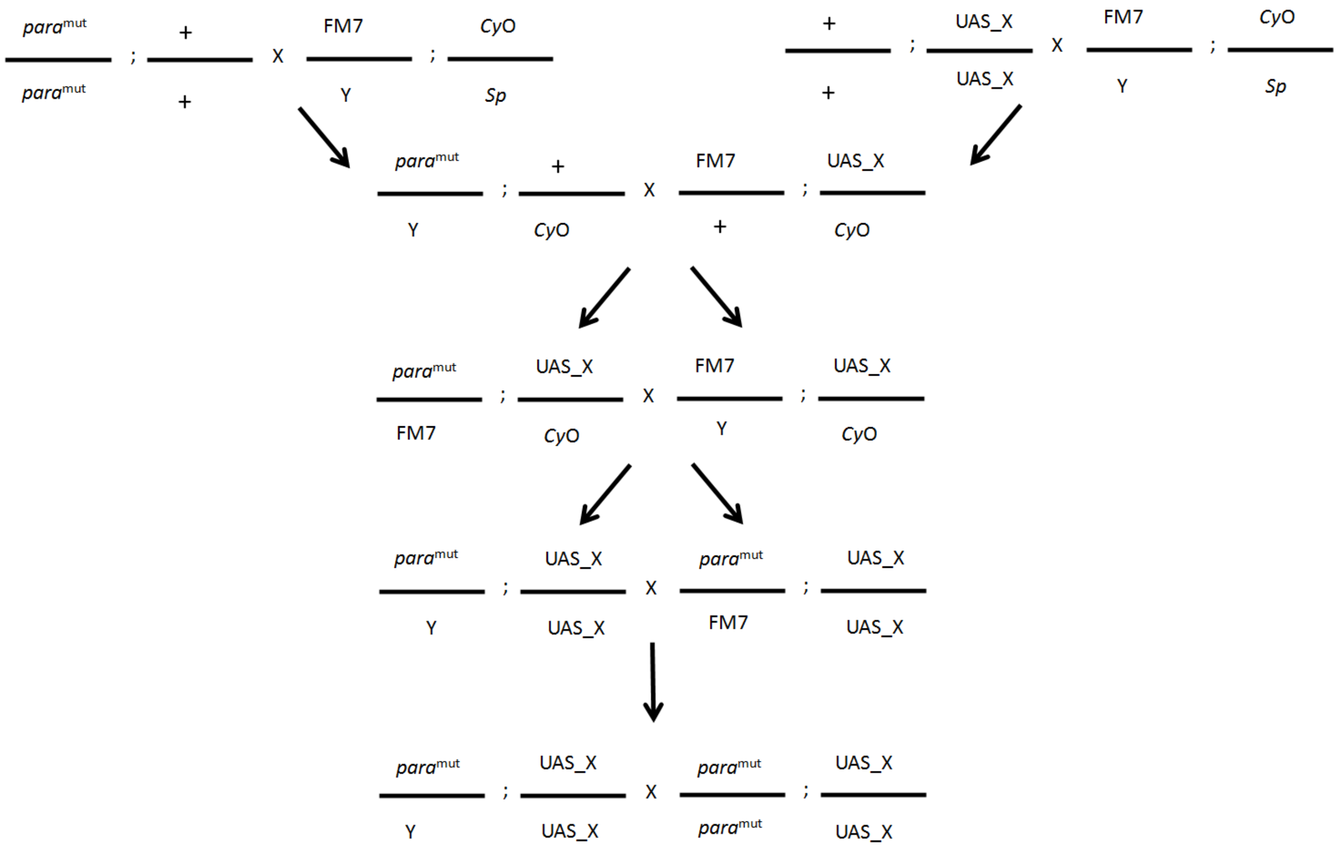

Figure S4: Crossing scheme for generation of *Drosophila* lines expressing P450s in mutant *para* genetic background. Genome engineered females bearing *para* mutations (*para*^{mut}) are crossed to FM7;*Cy*O/*Sp* males in order to generate *para*^{mut}; *Cy*O/+ males, while females from genetically transformed lines with a UAS insertion (UAS_X) are also crossed to FM7;*Cy*O/*Sp* males in order to generate FM7/+;UAS_X/*Cy*O females that are crossed to the *para*^{mut}; *Cy*O/+ males. Following the series of crosses depicted, we can generate strains bearing any transgene insertion located in chromosome 2 together with any X-linked *para* mutation of interest. In a similar fashion we have introduced both the HR-GAL4 driver and the UAS-CYP6BQ23 responder transgene in a *para*^{L1014F} genetic background, and the [HR-GAL4_UAS-CYP9J28] linked alleles in a *para*^{V1016G} genetic background.

Supplementary reference:

38. Ponton F, Chapuis MP, Pernice M, Sword GA, Simpson SJ. 2011. Evaluation of potential reference genes for reverse transcription-qPCR studies of physiological responses in *Drosophila melanogaster*. *J. Insect Physiol*. **57**, 840–850. (doi:10.1016/j. jinsphys.2011.03.014)
